# In correspondence to “Spontaneous common bile duct perforation in full term pregnancy: a rare case report and review of literature”

**DOI:** 10.1186/s12893-022-01512-3

**Published:** 2022-02-22

**Authors:** Goran Augustin

**Affiliations:** School of Medicine University of Zagreb, Department of Surgery, University Hospital Centre Zagreb, Kišpatićeva 12, 10000 Zagreb, Croatia

**Keywords:** Common bile duct, Perforation, Pregnancy, Incidence

## Abstract

**Aim:**

The correspondence letter aims to correct the historical perspective on common bile duct perforations (CBD) during pregnancy and complete the number of published cases.

**Findings:**

Instead of declared article by Piotrowski et al., from 1990, and according to available English language literature, the first two descriptions of maternal spontaneous CBD perforation in pregnancy were by JT Hogan Jr in 1957 and then Maurice Abitbol in 1958. Additional six cases of this condition were found, which is an increase of 50% of published cases.

**Purpose:**

The purpose of this correspondence letter is to correct the historical perspective on CBD perforation during pregnancy. Also, only all published cases in English language literature can shed new light on incidence, diagnosis, treatment, and maternal and fetal prognosis from maternal CBD perforation in pregnancy.

**Dear Editor,**

With great attention and interest, I read the case report by Masroor and Sarwari [1]. They aimed to present an extremely rare case of spontaneous common bile duct (CBD) perforation during pregnancy.

I find it very important to publish such rare cases, particularly during pregnancy, to understand how to deal with these conditions. Another importance of publishing rare cases of the same pathology during pregnancy is that it gives us insight into incidence, pathophysiology, specific clinical presentation, the accuracy of diagnostic methods, treatment methods with best outcomes, and maternal and fetal prognoses related to these conditions. Therefore, I congratulate the authors for this detailed case presentation with an excellent intraoperative photograph of CBD perforation.

Unfortunately, I believe there are several flows in the article. The authors collected cases with CBD and gallbladder rupture/perforation. Gallbladder rupture is far more common in the general population, probably due to different pathophysiology. This entity should be analyzed and described separately. Despite the gallbladder perforation analysis, even these perforations during pregnancy are lacking. Wade Stone published the first case in pregnancy in English literature [2] in 1937. Masroor and Sarwari should concentrate on CBD perforations in pregnancy and puerperium. Also, ruptures of choledochal cysts in pregnancy have different pathophysiology and should be analyzed separately.

My second observation is that the authors mention the first published case (by John Freeland in 1882) [3] in the general population, although it is debatable whether the case was spontaneous. It was due to multiple diverticula containing stones along the CBD. The authors also mentioned the first published case in infancy. Indeed it is important to note the first cases. However, for the pregnant population, it is interesting to know the first published case in pregnancy, particularly spontaneous, which was the main topic of the study by Masroor and Sawari. Masroor and Sarwari erroneously attribute the first case to Piotrowski et al. from 1990 [4]. According to available English language literature, the first two descriptions of spontaneous CBD perforation in pregnancy were by JT Hogan Jr in 1957 [5] and then Maurice Abitbol. The latter was from the Jewish Hospital of Brooklyn in 1958 [6]. He was unaware of the article published by Hogan Jr. Interestingly, the report by McGrath et al., included in the review by Masroor and Sawari, stated that JT Hogan Jr published the first case in 1957.

My third observation concerns their further discussion of the disease, which is about the data from the general non-pregnant population. The authors state different percentages of CBD perforations from those found in their reference by Piotrowski et al. [4]. Piotrowski et al. state 5% perforations in the bile ducts, while Masroor and Sarwari claim 4.4% perforations of CBD and 1.1% of the common hepatic duct. Another important finding is that these were not original data from Piotrowski et al., who cited the reference by Clarence McWilliams from 1912 [7]. The data from McWilliams’s article are found in Masroor and Sarwari’s article. It would be advisable to include more recent studies with newer data on the subject for a more objective comparison.

Interestingly, Piotrowski et al. from 1990 [4] claim that their case was the first published case. They concluded “spontaneous”, but gallstone was the cause, and the perforation site was dominantly common hepatic duct, with extension to CBD. The site of perforation was not stated in Table 1 of the article by Masroor and Sarwari.

Masroor and Sawari write: “*About 70% of spontaneous biliary perforation cases were associated with gall stones where the stone were found during surgery”*, with the reference at the end. This is misleading because sentences before and after this statement are about the pregnant population. Unfortunately, this percentage is for the general population. There is no data for the pregnant population due to its extreme rarity.

Furthermore, the authors state: “*According to our analysis, the most common site of biliary tract perforation during pregnancy is gall bladder (9/15; 60%), CBD (5/15; 33.3%), and hepatic duct (1/15; 6.66%). The causes of spontaneous perforation include gall stones (6/9; 66.6%) and idiopathic (3/9; 33.3%).”*. Due to the higher number of CBD perforation cases and probably gallbladder perforation, these values and conclusions could be different. Then, the complete section: *“The presentation of the patient can be different from case to case because it can have both acute and insidious onset. Most patients having insidious onset (80%) may present with abdominal distention without abdominal pain and clay color stool; progressive jaundice may follow [3, 6, 18]. In acute cases (20%), the signs and symptoms of the acute abdomen like generalized abdominal pain, abdominal distention due to bilious ascites, vomiting, fever, jaundice, high levels of bilirubin, or even shock may occur. The patient can present with a perihepatic collection or abscess instead of generalize peritonitis if the bile is localized to the area [3, 19].’*’ refers to articles from the general population. In Masroor and Sarwari’s Table 1., no data about clinical presentation exist. Therefore, we do not have clear evidence on symptoms, signs, and types of presentation.

Without further analysis, additional four references/cases (without JT Hogan Jr [5] and Maurice Abitbol [6]) of spontaneous CBD perforation are inserted here [8–11]. There is even a spontaneous hepatic duct perforation in pregnancy due to gallstones [12].

In the end, even the title could be changed to ‘’maternal spontaneous common bile duct perforation during pregnancy’’ to stress that condition occurred in a pregnant patient. This is important because many CBD perforations are found in infants.

In conclusion, there are two important issues with article publication and citations of other articles. First, scientific practice is to check on the authors/articles that accurately mentioned the term first, made the first diagnosis, or the first (successful) treatment of any disease. A thorough search is necessary even before the year 1900. The literature search should include keywords in other languages such as German, French, and Italian, which had great medicines of its time. Second, articles included as references should be read in full-text to confirm our statements or include statements from other articles with original data.

## Data Availability

No additional data.

## Abbreviation

CBD: Common bile duct

## References

[1] Masroor M, Sarwari MA (2021). Spontaneous common bile duct perforation in full term pregnancy: a rare case report and review of literature. BMC Surg.

[2] Stone WW (1937). Perforation of the gallbladder occurring in late stage of pregnancy. JAMA.

[3] Freeland J (1882). Rupture of the hepatic duct. Lancet.

[4] Piotrowski JJ, Van Stiegmann G, Liechty RD (1990). Spontaneous bile duct rupture in pregnancy. HPB Surg.

[5] Hogan JT (1957). Spontaneous rupture of the common bile duct during pregnancy. J Med Assoc Georg.

[6] Abitbol MM (1958). Rupture of the common bile duct as a cause of maternal death: a case report. Am J Obstet Gynecol.

[7] McWilliams CA (1912). Acute spontaneous perforation of the biliary system into the free peritoneal cavity. Ann Surg.

[8] Lemay M, Granger L, Verschelden G, Girard Y, Duguay L, Lavoie P (1980). Spontaneous rupture of the common bile duct during pregnancy. CMAJ.

[9] O’Neill A, O'Sullian MJ, McDermott E (2009). Spontaneous common bile duct rupture in a pregnant female—a rare cause of peritonitis. Eur J Obs Gynecol Reprod Biol.

[10] Thaggard WG, Johnson PN, Baron TH (1995). Endoscopic management of spontaneous bile duct perforation and bile peritonitis complicating term pregnancy. Am J Gastroenterol.

[11] Balsarkar DJ, Subramaniyan P, Joshi MA (2001). Spontaneous perforation of the common bile duct in pregnancy. Indian J Gastroenterol.

[12] Sauper T, Lanthaler M, Weissenboeck E, Nehoda H (2004). Spontaneous hepatic duct perforation in pregnancy: a case report and review of the literature. Gynecol Surg.

